# Supplementary material for: Generics in science communication: Misaligned interpretations across laypeople, scientists, and large language models
Source: Public Underst Sci. 2026 Apr 20;35(6):668–89. doi: 10.1177/09636625261425891 (PMC13380661; doi:10.1177/09636625261425891)
Supplement: sj-docx-1-pus-10.1177_09636625261425891 – Supplemental material for Generics in science communication: Misaligned interpretations across laypeople, scientists, and large language models [file sj-docx-1-pus-10.1177_09636625261425891.docx]

**Supplemental Material for**

**“Generics in science communication: Misaligned interpretations across laypeople, scientists, and large language models”**

Uwe Peters, Andrea Bertazzoli, Jasmine M. DeJesus, Gisela J. van der Velden, Benjamin Chin-Yee

**Table of contents**

1. Demographic details

2. Additional results of qualitative analyses

3. Additional methodological details

3.1 Power analysis

3.2 Material

3.3 LLM architectures and relevance

3.4 ‘Free response’ frame options

3.5 LLM data collection procedure

4. Additional research question and results

4.1 *RQ4*

4.2 Results for *RQ4*

4.3 Discussion of *RQ4* results

4.4 All preregistered hypotheses and related results

5. Linear mixed model details

**1. Demographic details**

| **Education (human participants)** | | | | | | | |
| --- | --- | --- | --- | --- | --- | --- | --- |
| **Discipline** | **High school, some college** | **BA, BSc** | **MA, MSc** | **PhD** | **MD, JD, DDS** | **Multiple (higher)** | **Total** |
| Clinical psychology | 0 (0%) | 2 (33.3%) | 2 (33.3%) | 2 (33.3%) | 0 (0%) | 0 (0%) | 6 |
| Cognitive psychology | 0 (0%) | 6 (30%) | 6 (30%) | 8 (40%) | 0 (0%) | 0 (0%) | 20 |
| Developmental psychology | 1 (1.3%) | 9 (12%) | 15 (20%) | 48 (64%) | 0 (0%) | 2 (2.7%) | 75 |
| Social psychology | 0 (0%) | 0 (0%) | 15 (38.5%) | 22 (56.4%) | 0 (0%) | 2 (5.1%) | 39 |
| Psychology (other) | 0 (0%) | 7 (33.3%) | 8 (38.1%) | 6 (28.6%) | 0 (0%) | 0 (0%) | 21 |
| Biomedical or health sciences | 0 (0%) | 23 (26.1%) | 25 (28.4%) | 17 (19.3%) | 13 (14.8%) | 10 (11.4%) | 88 |
| Social sciences (not psychology) | 1 (4.3%) | 16 (69.6%) | 5 (21.7%) | 1 (4.3%) | 0 (0%) | 0 (0%) | 23 |
| Natural sciences | 1 (3.7%) | 15 (55.6%) | 5 (18.5%) | 6 (22.2%) | 0 (0%) | 0 (0%) | 27 |
| Engineering or technology | 2 (4.1%) | 44 (89.8%) | 3 (6.1%) | 0 (0%) | 0 (0%) | 0 (0%) | 49 |
| Humanities | 0 (0%) | 27 (87.1%) | 4 (12.9%) | 0 (0%) | 0 (0%) | 0 (0%) | 31 |
| Other | 8 (15.1%) | 30 (56.6%) | 6 (11.3%) | 4 (7.5%) | 3 (5.7%) | 2 (3.8%) | 53 |
| Total | 13 (3%) | 179 (41.4%) | 94 (21.8%) | 114 (26.4%) | 16 (3.7%) | 16 (3.7%) | 432 |

**Table S1.** Participants’ education by discipline. “Multiple (higher)” refers to participants with more than one advanced degree (e.g., PhD and MD).

| **Discipline** | **Laypeople** | **Experts** | **ChatGPT-5** | **DeepSeek-V3.1** | **Total** |
| --- | --- | --- | --- | --- | --- |
| Psychology | 25 (15.5%) | 136 (84.5%) | 0 (0%) | 0 (0%) | 161 |
| Biomedical or health sciences | 23 (26.1%) | 65 (73.9%) | 0 (0%) | 0 (0%) | 88 |
| Other science | 79 (79.8%) | 20 (20.2%) | 0 (0%) | 0 (0%) | 99 |
| Other expertise | 65 (77.4%) | 19 (22.6%) | 0 (0%) | 0 (0%) | 84 |
| LLM | 0 (0%) | 0 (0%) | 50 (50%) | 50 (50%) | 100 |
| Total | 192 (36.1%) | 240 (45.1%) | 50 (9.4%) | 50 (9.4%) | 532 |

**Table S2.** Expertise by discipline. “Other sciences” included natural science, social science (not psychology), and engineering or technology. “Other expertise” included humanities scholars and “other” respondents. They were grouped together due to low sample size.

| **Demographics** | |
| --- | --- |
| **Country** |  |
| Austria | 5 (1.2%) |
| Belgium | 2 (0.5%) |
| Canada | 79 (18.3%) |
| France | 5 (1.2%) |
| Germany | 34 (7.9%) |
| Italy | 6 (1.4%) |
| Netherlands | 37 (8.6%) |
| Spain | 2 (0.5%) |
| UK | 58 (13.4%) |
| USA | 116 (26.9%) |
| Other | 88 (20.4%) |
| Total | 432 (100%) |
| **Gender** |  |
| Female | 256 (59.3%) |
| Male | 163 (37.7%) |
| Non-binary | 11 (2.5%) |
| Prefer not to say | 2 (0.5%) |
| **Native English speaker** |  |
| Yes | 277 (64.1%) |
| No | 155 (35.9%) |

**Table S3.** Participant demographics: country, gender, and English speaker status

**2. Additional results of qualitative analyses**

| **Expertise** | **No idea** | **‘XYZ is_’** | **‘suggests that_’** | **‘might_’** | **‘XYZ was_’** | **More than one choice** | **Total** |
| --- | --- | --- | --- | --- | --- | --- | --- |
| Laypeople | 29 (15.1%) | 12 (6.2%) | 106 (55.2%) | 22 (11.5%) | 21 (10.9%) | 2 (1%) | 192 |
| Human experts | 58 (24.2%) | 7 (2.9%) | 114 (47.5%) | 19 (7.9%) | 33 (13.8%) | 9 (3.8%) | 240 |
| ChatGPT-5 | 0 (0%) | 26 (52%) | 24 (48%) | 0 (0%) | 0 (0%) | 0 (0%) | 50 |
| DeepSeek-V3.1 | 0 (0%) | 2 (4%) | 28 (56%) | 0 (0%) | 20 (40%) | 0 (0%) | 50 |
| **Total** | 87 (16.4%) | 47 (8.8%) | 272 (51.1%) | 41 (7.7%) | 74 (13.9%) | 11 (2.1%) | 532 |
| **By discipline** |  |  |  |  |  |  |  |
| Psychology | 39 (24.2%) | 3 (1.9%) | 81 (50.3%) | 11 (6.8%) | 24 (14.9%) | 3 (1.9%) | 161 |
| Biomedicine | 20 (22.7%) | 3 (3.4%) | 43 (48.9%) | 8 (9.1%) | 11 (12.5%) | 3 (3.4%) | 88 |
| Other sciences | 16 (16.2%) | 4 (4%) | 53 (53.5%) | 10 (10.1%) | 12 (12.1%) | 4 (4%) | 99 |
| Other expertise | 12 (14.3%) | 9 (10.7) | 43 (51.2%) | 12 (14.3%) | 7 (8.3%) | 1 (1.2%) | 84 |

**Table S4.** Frequency and proportions of frame choices on the free response item.

| **Response** | **Laypeople** | **Human experts** | **ChatGPT-5** | **DeepSeek-V3.1** | **Total** |
| --- | --- | --- | --- | --- | --- |
| No reason | 83 (43.2%) | 121 (50.4%) | 0 (0%) | 0 (0%) | 204 (38.3%) |
| Only theme (1) | 49 (25.5%) | 37 (15.4%) | 0 (0%) | 0 (0%) | 86 (16.2%) |
| (1) and (2) | 12 (6.2%) | 31 (12.9%) | 6 (12%) | 9 (18%) | 58 (10.9%) |
| (1), (2), and (3) | 2 (1%) | 9 (3.8%) | 18 (36%) | 31 (62%) | 60 (11.3%) |
| (1) and (3) | 5 (2.6%) | 7 (2.9%) | 7 (14%) | 0 (0%) | 19 (3.6%) |
| (2) | 23 (12%) | 25 (10.4%) | 0 (0%) | 0 (0%) | 48 (9%) |
| (2) and (3) | 3 (1.6%) | 2 (0.8%) | 0 (0%) | 7 (14%) | 12 (2.3%) |
| (3) | 15 (7.8%) | 8 (3.3%) | 19 (38%) | 3 (6%) | 45 (8.5%) |
| Total | 192 (100%) | 240 (100%) | 50 (100%) | 50 (100%) | 532 (100%) |
| **Response** | **Psychology** | **Biomedicine** | **Other sciences** | **Other expertise** | **Total** |
| No reason | 81 (50.3%) | 45 (51.1%) | 39 (39.4%) | 39 (46.4%) | 204 (38.3%) |
| Only theme (1) | 24 (14.9%) | 17 (19.3%) | 27 (27.3%) | 18 (21.4%) | 86 (16.2%) |
| (1) and (2) | 20 (12.4%) | 10 (11.4%) | 8 (8.1%) | 5 (6%) | 67 (12.6%) |
| (1), (2), and (3) | 6 (3.7%) | 1 (1.1%) | 3 (3.0%) | 1 (1.2%) | 40 (7.5%) |
| (1) and (3) | 5 (3.1%) | 2 (2.3%) | 4 (4.0%) | 1 (1.2%) | 29 (5.5%) |
| (2) | 18 (11.2%) | 10 (11.4%) | 9 (9.1%) | 11 (13.1%) | 48 (9%) |
| (2) and (3) | 2 (1.2%) | 0 (0%) | 3 (3%) | 0 (0%) | 6 (1.1%) |
| (3) | 5 (3.1%) | 3 (3.4%) | 6 (6.1%) | 9 (10.7%) | 52 (9.8%) |
| Total | 161 (100%) | 88 (100%) | 99 (100%) | 84 (100%) | 532 (100%) |

**Table S5**. Frequency and proportions of reasons for frame choices on the free response item. Theme (1) avoiding extremes, (2) relativization to source study, and (3) informativity concerns

**3. Additional methodological details**

3.1 Power analysis

Input parameters: *F* tests, ANOVA: Fixed effects, omnibus, one-way, A priori, Effect size *f*: 0.25, α err prob: 0.05, Power (1–β): 0.80, Number of groups: 7.

3.2 Material

The first set of 9 generics included 6 generics about interventions, drugs, or diseases (e.g., ‘EV71vac is safe.’) and 3 about people (e.g., ‘Autistic people have longer daily screen use.’). The second set included 6 about people and 3 about interventions, drugs, diseases, etc. Of the 18 hedged conclusions, 6 used the modal verb ‘might’ and 12 the frame ‘suggest that [generic]’. The rationale was that ‘might’ statements are compatible with both significant and non-significant results, whereas ‘suggest that’ indicates a statistically positive finding. The ‘suggest that’ format thus provided a clearer contrast with bare generics, which assert claims directly.

3.3 LLM architectures and relevance

The two LLMs included in this study, ChatGPT-5 and DeepSeek-V3.1, differ in their underlying architectures (Rahman et al., 2025). DeepSeek-V3.1 uses a “mixture-of-experts” (MoE) design. In this approach, only a small subset of specialized parameter groups (“experts”) is activated for each input. Different subsets may be engaged on different runs, allowing the model to distribute workload across experts but also introducing greater variability in the resulting outputs. By contrast, ChatGPT-5 relies on a standard dense transformer architecture, in which all parameters are active for every input. This design can provide more uniform processing across runs, with performance further refined through reinforcement learning from human feedback.

These architectural differences may plausibly shape how the models produce science summaries. First, dense transformers may generate more stable summaries, while MoE systems could produce more diverse but less predictable ones. Second, MoE models route inputs through subsets of ‘experts’ that may capture particular domains (e.g., biomedical vs. social science), potentially supporting domain-specific detail but at the cost of consistency across fields. Dense transformers integrate all parameters, which may yield broader but more generalized summaries. Third, since only part of the model is used on each input, MoE responses may leave some available knowledge untapped in a given output, whereas dense models consistently mobilize the full parameter set. Finally, ChatGPT-5 has undergone extensive reinforcement learning from human feedback, which may make its summaries clearer and more user-oriented than those from MoE systems. Hence, including both architectures in our analyses allows us to test whether observed effects generalize across models that differ in how they process information.

3.4 ‘Free response’ frame options

(1) “XYZ is an effective treatment.” (“People with feature F experience E.”)

(2) “This study suggests that XYZ is an effective treatment.” (“This study suggests that people with feature F experience E.”)

(3) “XYZ might be an effective treatment.” (“People with feature F might experience E.”)

(4) “XYZ was an effective treatment.” (“People with feature F experienced E.”)

**3.4 LLM data collection procedure**

ChatGPT-5 and DeepSeek-V3.1 received the same survey as human participants. To ensure comparability, the model was sequentially prompted one conclusion and its three associated questions at a time, using the prompt:

“I will now survey you on your views regarding the interpretation of scientific conclusions. You will see 18 different one-sentence conclusions summarizing findings from published scientific studies, presented one at a time. After each conclusion, you will be asked three questions. Please read each conclusion carefully and then provide what you yourself think is the right response. Assume that all reported results are equally statistically significant and have the same effect size. How to respond: Use the provided 1–5 options exactly as labeled for each question (e.g., provide the numerical value behind your choice). Complete the free-response item that will be shown at the end.”

All LLM responses are available on our OSF platform.

**4. Additional research question, hypotheses, and results**

4.1 RQ4

A fourth research question was preregistered:

*RQ4.* Among experts in psychology and biomedicine, do the effects of generics on perceived generalizability, credibility, or impact vary by discipline depending on whether the conclusions concern biomedical or psychological findings?

Due to space reasons, the analysis and results are not reported in the main text but mentioned here.

To test *RQ4*, a third model was conducted, focusing only on psychologists and biomedical researchers and their understanding of generics (other frames were excluded). It included expertise (2 levels – psychologists, biomedical researchers), English speaker status, conclusion field, research years, research percentage (of work time), and the interaction between expertise and conclusion field.

4.2 Results for RQ4

*Generalizability.* In the model with psychologists and biomedical conclusion as the references, conclusion field had a significant effect with psychologists rating psychological generics (*M* = 3.01, *SE* = 0.11) as less generalizable than biomedical ones (*M* = 3.34, SE = 0.11, *b* = −0.25, *SE* = 0.11, *p* = 0.04). While the interaction between expertise and conclusion field was not significant (*p* = 0.24), in the planned comparison, biomedical researchers showed a descriptively similar pattern (psychological generics *M* = 2.83, *SE* = 0.148, biomedical generics *M* = 3.02, *SE* = 0.15).

*Credibility.* Psychologists rated psychological conclusions (*M* = 2.67, *SE* = 0.12) as less credible than biomedical ones (*M* = 3.15, SE = 0.12, *b* = −0.52, *SE* = 0.15, *p* = 0.003). However, there was no significant interaction between expertise and conclusion field, *F*(1, 825.1) = 0.99, *p* = 0.32.

*Impact.* Psychologists rating biomedical generics (*M* = 2.83, *SE* = 0.10) as more impactful than psychological ones (*M* = 2.65, *SE* = 0.10, *b* = −0.45, *SE* = 0.10, *p* < 0.001). Moreover, a significant interaction between expertise and conclusion field, indicated that this favouring of biomedical (*M* = 3.10, *SE* = 0.12) over psychological conclusions (*M* = 2.39, *SE* = 0.13) was even larger for biomedical researchers (*b* = −0.52, *SE* = 0.11, *p* < 0.001).

4.3 Discussion of RQ4 results

For generalizability and credibility, psychologists rated psychological generics lower than biomedical ones – a pattern consistent with the idea that field-specific background facilitates a narrower interpretation of field-specific generics (Coon et al., 2021). However, the interaction with expertise was not significant. For impact, the interaction between expertise and conclusion field was significant. *Both* groups rated biomedical generics as more impactful than psychological generics, and this preference for biomedical over psychological claims was even stronger for biomedical researchers, who did not downgrade their own field’s generics. These results seem to contradict the prediction that scientists rate generics from their own fields more narrowly (across domains) than those from other fields because of either increased epistemic vigilance or common ground assumptions (implicit qualifiers, etc.).

However, one possible explanation consistent with the *epistemic vigilance account* is that the replication crisis was especially pronounced in psychology (Baker, 2016) and many psychological topics show greater contextual sensitivity (e.g., cultural variation) than biomedical topics (van Bavel et al., 2016). Awareness of these factors may both increase greater “intellectual humility” among psychologists (Hoekstra and Vazire, 2021), prompting narrower interpretations of their field’s generics, while also prompting informed field outsiders, including biomedical researchers, to be more sceptical of psychological generics, in particular. Relatedly, biomedical researchers might not see a need to downgrade claims within their own field compared to psychology due to a perceived difference in “epistemic cultures,” with biomedical research being viewed as more rigorous, more routinely using randomized controlled trials to identify causal mechanisms, than psychological research (Albert et al., 2008).

4.4 All preregistered hypotheses and related results

Due to space constraints, we focused only on our three main hypotheses in the main text. Here we provide the complete list of preregistered hypotheses (H1–H6) together with the corresponding results.

*H1. Scientific conclusions presented in bare generic form will be rated as more generalizable, credible, and impactful than their past-tense or hedged versions, across all groups.*

Result. Supported in part. Reported in the main text.

*H2. Laypeople will interpret generics more broadly than scientists and LLMs, providing higher generalizability, credibility, and impact ratings.*

Result. Partially supported. Reported in the main text.

*H3. Scientists will show less variation in their responses across different linguistic framings compared to laypeople and LLMs.*

Result. Not supported. Reported in the main text.

*H4. Hedged and past-tense versions will elicit higher credibility ratings than bare generics.*

Note that H4 refers to scientists. Result. Partially supported. See main text and Table 2.

*H5. The effect of linguistic framing (generic, past, hedged) and disciplinary expertise on generalizability, credibility, or action relevance ratings will differ depending on the content of the claim (e.g., whether the research claim refers to people or abstract phenomena, to biomedical vs. psychological research, etc.).*

Result. Supported. See above.

*H6. DeepSeek-V3.1 will differ from humans in the same direction as ChatGPT-5 on the preregistered outcome(s), consistent with the interpretation that the effect may reflect general properties of popular contemporary LLMs rather than an idiosyncrasy of ChatGPT-5.*

Result. Supported. See main text. Table 2.

**5. Linear mixed model details**

Below are the details of the two main models used for the analyses reported in the main text.

| **Model** | **Outcome** | **Marginal R²** | **Conditional R²** | **Fixed effects (df)** | **N observations** | **N participants** | **N claims** |
| --- | --- | --- | --- | --- | --- | --- | --- |
| Model 1 | Generalizability | 0.080 | 0.326 | 10 | 8618 | 532 | 18 |
| Model 1 | Credibility | 0.187 | 0.428 | 10 | 8618 | 532 | 18 |
| Model 1 | Impact | 0.077 | 0.408 | 10 | 8618 | 532 | 18 |
| Model 2 | Generalizability | 0.089 | 0.335 | 22 | 8618 | 532 | 18 |
| Model 2 | Credibility | 0.189 | 0.430 | 22 | 8618 | 532 | 18 |
| Model 2 | Impact | 0.079 | 0.410 | 22 | 8618 | 532 | 18 |

**Table S6.** Model fit summary for linear mixed models. *Note.* Marginal R² reflects variance explained by fixed effects; conditional R² reflects variance explained by fixed and random effects combined. The difference between marginal and conditional R² indicates substantial clustering by participants, which is typical for repeated-measures judgment data and motivates the use of linear mixed-effects models. All models showed significant improvement over intercept-only models (likelihood ratio tests, *ps* < .001). For the variables included per model, see the main text.

| **Fixed effects omnibus tests of model 1** | | | | |
| --- | --- | --- | --- | --- |
| **Outcome** | **Effect** | **F** | ***df*** | ***p*** |
| Generalizability | Frame | 109.83 | 2 | < .001 |
|  | Expertise | 17.24 | 6 | < .001 |
| Credibility | Frame | 45.35 | 2 | < .001 |
|  | Expertise | 53.54 | 6 | < .001 |
| Impact | Frame | 4.74 | 2 | .009 |
|  | Expertise | 10.76 | 6 | < .001 |

**Table S7.** *Note.* English speaker status and claim type were included as covariates but are omitted here for brevity; neither altered the pattern of results (full output available upon request).

| **Fixed effects omnibus tests of model 2** | | | | |
| --- | --- | --- | --- | --- |
| **Outcome** | **Effect** | ***F*** | ***df*** | ***p*** |
| Generalizability | Frame | 56.15 | 2 | < .001 |
|  | Expertise | 17.21 | 6 | < .001 |
|  | Frame * expertise | 9.38 | 12 | < .001 |
| Credibility | Frame | 30.38 | 2 | < .001 |
|  | Expertise | 53.55 | 6 | < .001 |
|  | Frame * expertise | 2.36 | 12 | .005 |
| Impact | Frame | 2.25 | 2 | .106 |
|  | Expertise | 10.76 | 6 | < .001 |
|  | Frame * expertise | 2.15 | 12 | .012 |

**Table S8.** Details of Frame*expertise interaction. English speaker status and claim type were again included as covariates but are omitted here for brevity; neither altered the pattern of results (full output available upon request).

| **Random effects variance components and intraclass correlations** | | | | | |
| --- | --- | --- | --- | --- | --- |
| **Model** | **Outcome** | **Random effect** | **Variance** | ***SD*** | **ICC** |
| Model 1 | Generalizability | Individual ID | 0.302 | 0.550 | 0.236 |
|  |  | Claim ID | 0.055 | 0.234 | 0.053 |
| Model 1 | Credibility | Individual ID | 0.198 | 0.445 | 0.241 |
|  |  | Claim ID | 0.065 | 0.254 | 0.094 |
| Model 1 | Impact | Individual ID | 0.352 | 0.594 | 0.335 |
|  |  | Claim ID | 0.039 | 0.197 | 0.052 |
| Model 2 | Generalizability | Individual ID | 0.302 | 0.550 | 0.238 |
|  |  | Claim ID | 0.054 | 0.233 | 0.053 |
| Model 2 | Credibility | Individual ID | 0.198 | 0.445 | 0.241 |
|  |  | Claim ID | 0.065 | 0.255 | 0.094 |
| Model 2 | Impact | Individual ID | 0.353 | 0.594 | 0.336 |
|  |  | Claim ID | 0.039 | 0.197 | 0.052 |

**Table S9.** Random-effects variance components and intraclass correlation coefficients (ICCs). *Note.* Individual ID and claim ID were simply unique identifying numbers to control for repeated measures per participant (individual ID) and variation in conclusion content (claim ID). ICCs quantify the proportion of variance attributable to clustering by participants and claims. Substantial participant-level ICCs and smaller but non-trivial claim-level ICCs justify the inclusion of random intercepts for both grouping factors.
